# Evaluation of Vitamin D Supplementation in Critically Ill Patients—A Narrative Review of Randomized Controlled Trials Published in the Last 5 Years

**DOI:** 10.3390/nu17050816

**Published:** 2025-02-27

**Authors:** Shan Wang, Ruodi Ren, Kunkun Wang, Christopher Leo, Mengyan Li, Allison Chow, Andrew K. Yang, Yun Lu

**Affiliations:** 1 NYU Langone Hospital—Long Island, Mineola, NY 11501, USA; shan.wang@nyulangone.org; 2 College of Pharmacy, University of Minnesota, Minneapolis, MN 55455, USA; renruodicc@gmail.com; 3 Fairbanks Memorial Hospital, 340 Cowles Street, Fairbanks, AK 99701, USA; vector0318@gmail.com; 4 Duke Raleigh Hospital, a Campus of Duke University Hospital, School of Medicine, Duke University, Durham, NC 27708, USA; christopher.leo@duke.edu; 5 Brigham and Women’s Hospital, Boston, MA 02115, USA; meli@bwh.harvard.edu; 6 College of Arts and Science, New York University, New York, NY 10012, USA; ayc8022@nyu.edu; 7 Dartmouth College, Hanover, NH 03755, USA; andrew.k.yang.21@dartmouth.edu; 8 Department of Pharmacy, Hennepin Healthcare System, Minneapolis, MN 55415, USA

**Keywords:** clinical outcomes, vitamin D deficiency, vitamin D replacement, critically ill patients, vitamin D doses

## Abstract

The prevalence of vitamin D deficiency among intensive care unit (ICU) patients is potentially associated with an increased risk of mechanical ventilation, sepsis, prolonged hospital stays, and mortality. Although ICU patient care has significantly improved in recent years, the role of vitamin D supplementation remains under investigation. A literature review was conducted using PubMed, Web of Science, Embase, and Cochrane databases, focusing on randomized controlled trials published in the past five years on vitamin D supplementation in adult ICU patients. Patients’ baseline vitamin D levels, administration routes, doses, biomarker changes, mechanical ventilation duration, length of hospital stay, and mortality were analyzed. Although vitamin D supplementation appears safe and may reduce ICU stay duration and mechanical ventilation time and improve SOFA scores, its impact on overall mortality remains uncertain. Routine supplementation for all ICU patients is not currently recommended; clinical decisions should consider individual baseline vitamin D levels, patient characteristics, severity of illness, doses, and administration methods.

## 1. Introduction

It is reported that 40–70% of intensive care unit (ICU) patients present with vitamin D deficiency [1]. Vitamin D (calciferol) is a fat-soluble vitamin synthesized in the skin through UV exposure and obtained from food or dietary supplements. It undergoes hydroxylation in the liver to form 25-hydroxyvitamin D (calcidiol or calcifediol, supplement analog D3/cholecalciferol) and is further hydroxylated in the kidney into active metabolite 1,25-dihydroxyvitamin D (calcitriol, supplement analog D2/ergocalciferol) [2]. Cholecalciferol, ergocalciferol, and calcitriol are commonly used for supplementation in clinical settings [3].

Previous studies have shown that vitamin D is essential for immune function, a necessity for critical illness recovery. Vitamin D is also known to regulate gene expression, cell proliferation, and apoptosis [4]. Additionally, it controls the proliferation of T and B cells, modulates immunoglobulin production, and decreases proinflammatory cytokine levels [1,4,5]. Vitamin D has also been shown to upregulate cathelicidin and other antimicrobial peptides, which are essential for immune defense in critically ill patients [6].

Vitamin D deficiency (25-hydroxyvitamin D < 20 ng/mL) leads to higher levels of inflammation in certain tissues, including nervous and lung tissues [7,8]. Studies found that vitamin D deficiency may also increase the risk of respiratory failure [9,10]. Vitamin D deficiency is found to be associated with sepsis, infection, and increased morbidity and mortality [10]. Studies evaluating the efficacy of vitamin D replacement in critically ill patients have demonstrated conflicting results. This review aims to investigate the latest literature on the effects of vitamin D replacement in critically ill adult patients with regard to clinical outcomes while also evaluating respective vitamin D dosages.

## 2. Materials and Methods

**Figure 1:**
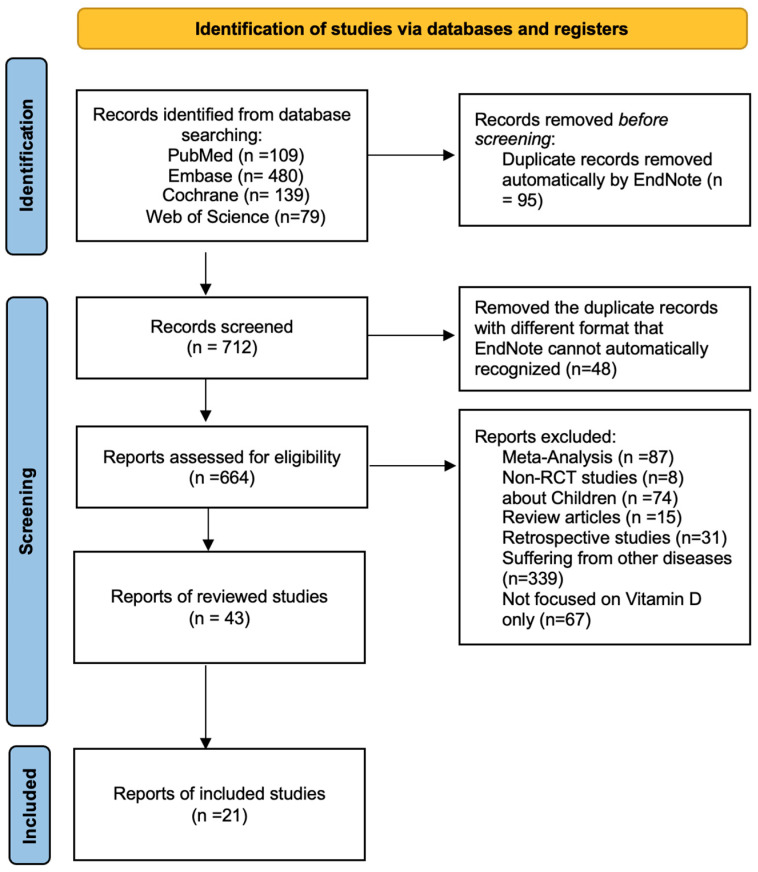
Preferred Reporting Items for Systematic Reviews and Meta-Analyses (PRISMA) flow diagram of study selection based on inclusion and exclusion criteria [11].

Two reviewers independently screened four databases (PubMed, Embase, Cochrane, and Web of Science) using predefined search terms—(vitamin D OR Cholecalciferol OR ergocalciferol OR calcitriol) AND (ICU OR intensive care OR critically-ill)—to identify human studies meeting all four of the following criteria: (1) the study design had to be a randomized controlled clinical trial (RCT); (2) the study had to be performed in an adult population (age ≥ 18 years); (3) the studies must have been published between 2019 and November 2024; (4) the full-length publications must have been available in the English language; and (5) vitamin D was the only variable studied in the clinical outcomes. Retrospective studies, observational studies, and meta-analyses were excluded (see Figure 1).

Detailed data were extracted from each trial, including baseline vitamin D levels, patient population characteristics, sample size analyzed, vitamin D replacement routes/doses, duration of replacement, dose duration, and study outcomes.

In this review, the term vitamin D level refers to serum 25-hydroxyvitamin D [25(OH)D] level.

Retrospective, observational, and meta-analysis papers were excluded. Table 1 presents baseline vitamin D status, patient populations, dosing regimens, and reported outcomes. Given the heterogeneity in study designs, each eligible study was assessed qualitatively.

## 3. Results

Out of 807 studies identified, 21 studies met the criteria and were included in this narrative review (Figure 1). For each included study, study details were extracted into Table 1. Two authors independently verified the extracted data for all studies. Eight of the 21 studies included did not show clinical benefits of vitamin D replacement in critically ill patients. The remaining 13 studies demonstrated that vitamin D replacement in ICU patients had a positive impact on certain clinical outcomes. Six of these 13 trials reported that vitamin D supplementation led to a decreased ICU or hospital length of stay (LOS), three trials showed an improvement in Sequential Organ Failure Assessment (SOFA) scores, three trials found a decrease in mechanical ventilation (MV) duration, three trials revealed a decrease in 30-day mortality, two trials demonstrated positive outcomes in the Glasgow Coma Scale (GCS), and six trials reported positive biomarker results. Table 1 summarizes the key findings of the trials included in this narrative review. Table 2 highlights the positive versus negative clinical outcomes following vitamin D replacement.

Various dosing replacement strategies were adopted in these 21 studies. Trials that showed a benefit of vitamin D replacement reported the following doses: oral 50,000 International Units (IU) daily for 5 days; oral 120,000 IU single dose, oral 600,000 IU single dose, and IM dose 300,000 IU single dose.

## 4. Discussion

Several meta-analyses and systematic reviews have examined the role of vitamin D supplementation in critically ill patients over the past five years. However, most meta-analyses were completed in or before 2022 [25,26,27,28,29,30,31]. Since then, multiple randomized controlled trials (RCTs) have been conducted, which we have included in our review.

Furthermore, our study categorizes clinical outcomes into three groups: positive impact, negative impact, and no impact of vitamin D supplementation in this patient population. Additionally, we compared different dosing strategies used in these RCTs to determine which dosages were most commonly administered and which were associated with the most positive clinical outcomes.

Overall, these studies on vitamin D replacement in ICU patients demonstrated conflicting results over the past 5 years. The VIOLET trial conducted by The National Heart, Lung, and Blood Institute’s PETAL clinical trial group (2019) showed that early enteral administration of a single 540,000 IU vitamin D3 high dose in critically ill patients with vitamin D deficiency (25-hydroxyvitamin D level < 20 ng/mL) increased vitamin D serum levels but did not demonstrate any clinical benefit over the placebo in terms of 90-day mortality or other nonfatal outcomes [8]. The authors conducted an ancillary study, VIOLET Long-term Brain Outcomes in Vitamin D Deficient Patients (VIOLET-BUD) [17]. The subsequent study evaluated the same single enteral high dose vitamin D3 versus placebo in patients with vitamin D deficiency, focusing on their long-term cognitive outcomes at a median follow-up of 443 days (interquartile range: 390–482 days). Long-term cognitive outcomes were evaluated using the Repeatable Battery for the Assessment of Neuropsychological Status (RBANS) [32], while executive function was assessed using a composite score derived from three subscales of the Delis–Kaplan Executive Function System (D-KEFS) [33]. It concluded that a single high dose of enteral vitamin D did not improve long-term global cognition or executive function in critically ill, vitamin D-deficient patients. Akbas et al. showed that vitamin D replacement had a positive impact on biomarkers such as procalcitonin, cathelicidin, LL-37, neutrophil-to-lymphocyte ratio (NLR), and platelet-to-lymphocyte ratio (PLR) levels [34]. Miri et al. demonstrated that vitamin D improved the SOFA score, GCS score, post-op infection rate, duration of mechanical ventilation, and ICU and hospital LOS [24]. Ingels et al. found that vitamin D replacement in ICU patients did not increase serum 25-(OH)-D levels as much as expected [20]. This is partly because serum calcitriol concentrations are more tightly regulated than 25-(OH)-D concentrations. Additionally, although 25-(OH)-D undergoes a second hydroxylation in the kidneys to produce circulating calcitriol for bone and muscle, it is also known to be activated in vitamin D-dependent tissues, including pancreatic islets and immune system cells. It is speculated that CYP27B1 (also known as 1-alpha hydroxylase) is downregulated during critical illness, which compromises the conversion of 25-(OH)-D to 1,25(OH)_2_D, and possibly shifts the metabolization of 25-(OH)-D to other compounds. A small increase in 24, 25 (OH)_2_D was also noticed, which might serve as a feedback mechanism for avoiding vitamin D toxicity [35]. Critical illness lowers serum 25-(OH)-D concentrations, though the underlying mechanisms remain unclear. Therefore, analyses using baseline 25-(OH)-D values—those measured from admission samples—are preferable. However, none are entirely free from this confounder, as patients requiring critical care are typically severely ill or have undergone major surgery. The vitamin D supplementation dose in the study by Ingels et al. was very low (loading dose of 8000 IU and daily maintenance of 600 IU × 10 days) compared to vitamin D doses in other studies. This could also explain why Ingels et al. did not find any significant benefit of vitamin D replacement in ICU patients.

The dosing and delivery strategies for vitamin D varied across studies and may be specific to institutional protocols. In studies that showed vitamin D replacement with positive clinical outcomes, the dose ranged from 5000 IU to 540,000 IU as a single dose or multiple-day replacement, given either via the enteral or the intramuscular route. Kearns et al. found significant changes in biochemical markers when evaluating a single dose of 600,000 IU of vitamin D_3_, highlighting the need for greater caution when administering single doses exceeding 500,000 IU [35]. Fortunately, no toxicity from vitamin D supplementation was reported in patients in all the studies reviewed.

The role of vitamin D in patients with severe vitamin D deficiency has been identified and confirmed in several studies [5,7,10,12,13,14,15,16,17,18,19,20,21]. In VITdAL-ICU, the use of vitamin D supplementation did not show beneficial effects on hospital or ICU LOS, hospital mortality, or 90-day mortality. A post-hoc subgroup analysis of patients with severe vitamin D deficiency (defined as vitamin D level ≤ 12 ng/mL) in the VITDAL-ICU study identified a significant reduction in 28-day mortality (HR 0.52, [0.30–0.89]) [36]. Similarly, Bhattacharyya et al. showed that vitamin D replacement significantly decreased the need for mechanical ventilation and trended toward reducing 90-day mortality (HR 0.449, [0.198–1.017]) [4]. VITDALIZE is designed to enroll 2400 patients, with a primary endpoint of 28-day mortality [36]. The outcome of this trial is expected to be reported in 2026 and may provide us with more robust evidence of the efficacy of vitamin D supplementation in patients with severe vitamin D deficiency.

Thampi et al., Sistanzid et al., and Naguib et al. used synthetic vitamin D analogs as a vitamin D replacement form [3,6,18]. They used calcitriol in septic patients and found no benefit of vitamin D replacement. Naguib et al. administered alfacalcidol orally to patients scheduled for elective mechanical valve replacement surgery [18]. Although Naguib et al. showed no significant difference in hospital mortality, there was a significant reduction in ICU length of stay and postoperative infection rate. Generally, vitamin D analogs were not suitable for vitamin D replacement due to their narrow therapeutic range and lack of feedback control, resulting in an increased risk of hypercalcemia. Analogs are indicated for hypocalcemia, osteoporosis, and the prevention of corticosteroid-induced osteoporosis [36]. Given the high incidence of acute kidney injury in acute illness and the high level of monitoring in ICUs, it is arguable that active or partially active vitamin D analogs are more suitable for the critically ill population. Further studies should be designed to evaluate their efficacy and safety in this context.

The role of vitamin D in critically ill COVID-19 patients was explored in several studies. A search using the keywords “calcifediol”, “COVID”, and “critically ill” identified only one randomized controlled trial (RCT): Entrenas Castillo et al. (2020) [37]. However, this study was conducted at a time when COVID-19 treatment options were poorly understood and included hydroxychloroquine, which was later found to have no therapeutic effect against COVID-19. Due to the inclusion of hydroxychloroquine in the treatment protocol, we deemed this study invalid and excluded it from our review.

Other research studies on vitamin D in COVID-19 patients had significant limitations. Bishop et al. (2022) [38] did not investigate its effects on critically ill COVID-19 patients, while De Niet et al. (2022) [39] did not focus on ICU patients in their analysis. Maghbooli et al. (2022) [40] reported that calcifediol supplementation was associated with an increased percentage of lymphocytes and a decreased neutrophil-to-lymphocyte ratio. A lower neutrophil-to-lymphocyte ratio was significantly linked to reduced ICU admission duration and mortality. However, since the study’s primary outcome was not a direct clinical endpoint, it was not included in our literature review.

Masbough et al. and Sharma et al. both investigated vitamin D replacement in traumatic brain injury patients [7,13]. Surprisingly, both studies found a statistically significant increase in GCS scores. Sharma et al. reported improvement in biomarkers and shorter mechanical ventilation days. Similarly, Hansaloei et al. reported a significant reduction in biomarkers, SOFA scores, duration of mechanical ventilation days, and length of ICU stay in critically ill patients with traumatic injuries [22]. The impact of preoperative vitamin D supplementation was examined by Hajimohammadebrahim-Ketabforoush et al. in patients undergoing craniotomy for brain tumor resection and by Naguib et al. in those undergoing elective mechanical valve replacement surgery. Both studies reported a statistically significant reduction in ICU length of stay [18,19]. Low vitamin D level was associated with adverse outcomes with various surgical procedures. With a relatively low patient population enrolled in these trials, the positive findings regarding GCS scores and reduced ICU length of stay suggest that this area could benefit from further exploration with larger patient enrollment.

The root cause of vitamin D deficiency in ICU patients can be attributed to both a pre-existing deficiency and a decline in levels during acute illnesses [41,42]. Mechanisms contributing to reduced vitamin D levels during acute illness may include hemodilution, interstitial extravasation, decreased synthesis of binding proteins, and renal loss [43]. In addition, acute fluid resuscitation in the ICU can significantly lower vitamin D levels, which may take up to 24 h to resolve [44]. Thus, interpretations of vitamin D levels in patients with acute illnesses should be performed with caution.

Absorption of vitamin D supplementation can vary in different patient populations. For example, higher BMI is linked to a smaller increase in serum 25(OH)D concentrations. Obesity reduces 25(OH)D responses to vitamin D treatment not only due to increased dilution of available 25(OH)D but also because it specifically decreases the activity of 25-hydroxylase in the liver and other tissues responsible for 25(OH)D production. Obesity is associated with the repression of the enzyme CYP2R1, which is primarily responsible for 25-hydroxylation of vitamin D in the liver, leading to decreased serum levels of 25(OH)D (the active form of vitamin D) due to reduced production of this metabolite; essentially, obesity inhibits the key step in the activation of vitamin D, resulting in lower circulating levels of the active form [45,46].

Calcium intake and the type of vitamin D (D2-ergocalciferol or D3-cholecalciferol) can affect the dose–response of 25(OH)D to vitamin D. Compared to ergocalciferol, cholecalciferol has better biological efficacy in improving vitamin D status [47]. Following oral intake, vitamin D is rapidly absorbed, reaching the maximum level after around 24 h. The levels of 25(OH)D increase gradually, peaking at 7–14 days, depending on the dose. It is not known how the 25(OH)D concentrations were affected in studies that used only single-dose vitamin D supplementation. Patients with malabsorption issues, such as gastrectomy or bariatric patients, might need higher doses of vitamin D [44]. Lastly, baseline vitamin D levels reflect coexisting conditions, especially in critically ill patients, which could cause residual confounding effects when analyzing results [8].

Given the wide therapeutic index of vitamin D, clinicians may feel comfortable with high-dose vitamin D replacement even in the absence of a baseline vitamin D level. However, vitamin D toxicity may occur when serum levels of 25(OH)D are greater than 150 ng/mL, accompanied by normal or elevated values of 1,25(OH)2D concentration. The most common cause of vitamin D toxicity is excessive vitamin D supplementation without frequent monitoring of vitamin D levels. While most cases of vitamin D toxicity do not lead to serious complications or sequelae, it may cause hypercalcemia and acute renal failure, which are important considerations in critical care settings. If a high-dose vitamin D replacement is given, it is reasonable to consider monitoring vitamin D levels together with electrolyte levels and kidney function. Studies have shown why large bolus doses of vitamin D can be ineffective, both for preventing rickets and reducing the risks of respiratory infections. This is because it induces increased 24-hydroxylation of circulating 25(OH)D, an effect that is normally protective against vitamin D toxicity. Furthermore, this adverse effect can last for a full month after a single large bolus dose of vitamin D [48]. In COVID-19 patients, larger bolus doses of vitamin D proved ineffective despite the known protective effects of hormonal vitamin D against infection—such as boosting innate immunity, downregulating excessive adaptive immune responses that trigger cytokine storms, and reducing the risk of acute respiratory distress syndrome (ARDS). One possible reason is that vitamin D_3_ takes approximately two weeks to raise low serum 25(OH)D levels to normal, whereas COVID-19 severity progresses more rapidly. Another potential explanation is that extremely high doses of vitamin D induce protective mechanisms against D_3_ toxicity, including increased activity of the 25-hydroxylase enzyme in the liver and other tissues; this, in turn, reduces the availability of 25(OH)D, the precursor required for the production of hormonal vitamin D (calcitriol) in tissues. Smaller bolus doses (<100,000 IU), however, may be more effective in COVID-19 patients [49,50,51,52,53]. Further studies are needed to determine the optimal dosing strategies for vitamin D supplementation in critically ill patients.

## 5. Limitations

This review has several limitations. First, no quantitative statistical analysis was conducted, which reduces certainty and introduces the potential for bias in the findings. Second, we did not perform a meta-analysis in our review. Previous meta-analyses have suggested that vitamin D supplementation may be associated with a reduced mortality rate and lower ICU admission rates [54].

Additionally, due to the heterogeneity of study designs and variability in vitamin D dosing, this review is limited by its approach of evaluating studies on an individual basis.

## 6. Conclusions

Vitamin D supplementation in critically ill patients may shorten ICU or hospital length of stay, improve SOFA scores, or reduce the duration of mechanical ventilation. Its ability to lower 28-day mortality remains uncertain, and large randomized controlled trials are needed to confirm its efficacy in this setting. Additionally, identifying the highest bolus dose of vitamin D that can be administered without triggering mechanisms that reduce its activation remains a critical research priority.

## Figures and Tables

**Table 1:** Summary of randomized controlled trials (RCTs) from 2019 to 2024.

Study	Year	Baseline Vit D Level (ng/mL)	Sample Size	Patient Population	Vit D Replacement Dose	Duration	Notes
Ashoor et al. [10]	2024	<20	80	Sepsis on mechanical ventilation	HD: enteral 50,000 IU/d vs. LD: enteral 5000 IU/d	5 days	Significant difference in procalcitonin, LL-37 reduction, improved SOFA, and hospital LOS
Singh et al. [12]	2024	12 vs. 13	90	COVID-19	Enteral 600,000 IU	Once	Significantly improved SOFA score at Day 7 and 28-day mortality
Masbough et al. [13]	2024	15.95 vs. 17.84	35	Traumatic brain injury	IM 300,000 IU	Once	Statistically significant increase in GCS scores, reduction in inflammatory markers, improvement in the GOS-E score; no difference in 28-day mortality, ICU LOS, MV needs
Thampi et al. [6]	2024	Not reported	152	Sepsis	Calcitriol IM 300,000 IU	Once	No significant difference in APACHE II scores, 28-day mortality, MV days, ICU LOS, and hospital-acquired infections
Sistanizad et al. [3]	2024	11.37 vs. 13.96	28	Sepsis	Calcitriol IV 1 mcg/day	3 days	No significant difference in procalcitonin level, ICU LOS, and 28-day mortality
Zamanian et al. [14]	2024	23.06 vs. 25.68	61	COVID-19	IM 300,000 IU	Once	No significant difference in mortality or hospital LOS
Wang et al. [15]	2024	<20	61	Vitamin D deficient	Enteral 569,600 IU	Once divided by 8 bottles	Only 41.5% of the patients achieved serum 25(OH)D levels higher than 30 ng/mL in the supplementation group. They had significantly lower 30-day mortality.
Domazet Bugarin et al. [16]	2023	25.3 vs. 27.3	155	COVID-19	Enteral 10,000 IU vs. placebo	Once	No statistical significance in MV days, secondary outcomes
Bychinin et al. [5]	2022	9.6 vs. 11	110	COVID-19	PO 60,000 IU weekly, then 5000 IU/daily	During hospital stay	Significantly increased NK and NK T cell counts. No difference in mortality, need for MV, or incidence of nosocomial infection; ICU and hospital LOS were longer in the vitamin D3 replacement group
Sistanizad et al. [1]	2021	<20	36	ICU ventilated	IM 300,000 IU vs. placebo	Once	No statistically significant results were identified due to the small sample size
Bhattacharyya et al. [4]	2021	12.05 vs. 15.47	126	Sepsis	Enteral 540,000 IU vs. placebo	Once	No statistical difference in ICU LOS, hospital LOS, MV duration/requirements, or 90-day mortality
Han et al. [17]	2021	15.2 vs. 13.1	95	Vitamin D deficient	Enteral 540,000 IU	Once	No significant difference in long-term global cognition or executive function
Naguib et al. [18]	2021	21 vs. 19.1	86	Elective mechanical valve replacement	Alfacalcidol 2 μg/day PO	Starting 2 days before surgery until the end of hospital stay	Statistically significant reduction in ICU LOS and postoperative infection rate. No significant difference in hospital mortality
Hajimohammadebrahim-Ketabforoush et al. [19]	2021	<20	60	Craniotomy for brain tumor resection	IM 300,000 IU	Once	Significant reduction in ICU LOS and hospital LOS
Sharma et al. [7]	2020	18.30 vs. 15.15	35	Traumatic brain injury	Enteral 120,000 IU vs. placebo	Once	Significant improvement in GCS score, MV duration, IL-6, and TNF-ɑ
Ingels et al. [20]	2020	6.8 vs. 9.2	24	Prolonged ICU stay (>10 days)	200 μg loading dose once, then 15 μg/day	Loading dose then 10 days	No difference in SOFA score or MV duration
Padhy et al. [21]	2020	≤20	60	Vitamin D-deficient, sepsis	G1: enteral 60,000 IU once/wk; G2: 60,000 IU twice/wk	During hospital stay	No difference was found in ICU LOS, duration of MV, and 28-day ICU mortality. Patients in group 2 required less inotropic support (*p* = 0.037)
Hasanloei et al. [22]	2020	10–30	72	Ventilated, traumatic injury	G1: PO 50,000 IU/day; G2: IM 300,000 IU vs. placebo	G1: 6 days; G2: once	Significant improvement in IL6, ESR, CRP, SOFA score, duration of MV, ICU LOS
Karsy et al. [23]	2020	14.6 vs. 13.9	267	Neurocritical care, vitamin D-deficient	PO 540,000 IU vs. placebo	Once	No statistical difference in hospital LOS or ICU LOS
Miri et al. [24]	2019	8.43 vs. 11.35	40	ICU ventilated	IM 300,000 IU vs. placebo	Once	Significant reduction in 28-day mortality
PETAL group [8]	2019	11.2 vs. 11.0	1078	Vitamin D- deficient	Enteral 540,000 IU vs. placebo	Once	No statistical difference in 90-day mortality and other clinical outcomes

**Table 2:** Summary of positive vs. negative clinical outcomes.

Year	Author	Clinical Results										Biomarkers
		ICU LOS	Hospital LOS	SOFA Score	MV Duration	MV Needs	90-Day Mortality	28-Day Mortality	30-Day Mortality	GCS	Less Inotropic Support	
2024	Ashoor et al. [10]		HD	HD								HD: pct, IL-37
2024	Singh et al. [12]											
2024	Masbough et al. [13]											IL-1b, IL-6
2024	Thampi et al. [6]											
2024	Sistanizad et al. [3]											pct
2024	Zamanian et al. [14]											
2024	Wang et al. [15]											
2023	Domazet Bugarin et al. [16]											
2022	Bychinin et al. [5]						All-cause					NK, NKT, CRP, pct
2021	Sistanizad et al. [1]											
2021	Bhattacharyya et al. [4]											
2021	Han et al. [17]									RBANS score		
2021	Naguib et al. [18]											
2021	Hajimohammadebrahim-Ketabforoush et al. [19]											
2020	Sharma et al. [7]											IL-6, TNF-α
2020	Ingels et al. [20]											CRP, WBC, IL-37, sCD163
2020	Padhy et al. [21]											
2020	Hasanloei et al. [22]											IL-6, ESR, CRP
2020	Karsy et al. [23]											
2019	Miri et al. [24]											
2019	PETAL group [8]											

Table 1 and Table 2: 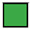: Vitamin D positively affected clinical outcomes; 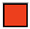: Vitamin D negatively affected clinical outcomes; 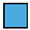: Vitamin D had no effect on clinical outcomes; 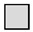: Not reported; HD: high dose of vitamin D (enteral 50,000 IU daily for 5 days) versus. low dose of vitamin D (enteral 5000 IU daily for 5 days); LOS: length of stay; APACHE II: acute physiologic assessment and chronic health evaluation II; SOFA: Sequential Organ Failure Assessment; MV: mechanical ventilator; All-cause: all-cause mortality; RBANS score: Repeatable Battery for the Assessment of Neuropsychological Status; GCS: Glasgow Coma Scale; IM: intramuscular injection; PO: by mouth; pct: procalcitonin; CRP: C-reactive protein; NK: natural killer cell; NKT: natural killer T cell; ESR: erythrocyte sedimentation rate; WBC: white blood cell; IL: interleukin; TNF-α: tumor necrosis factor-alpha; GOS-E: Glasgow outcome scale-extended.
